# Evaluation of Cervical Myoclonus in Dogs with Spinal Diseases: 113 Cases (2014–2023)

**DOI:** 10.3390/ani15152298

**Published:** 2025-08-06

**Authors:** Ana Martinez, Emili Alcoverro, Edward Ives, Lisa Alves

**Affiliations:** 1 Department of Veterinary Medicine, University of Cambridge, Madingley Road, Cambridge CB3 0ES, UK; la382@cam.ac.uk; 2 Department of Neurology and Neurosurgery, ChesterGates Veterinary Specialists, Chester CH1 6LT, UK; emili.alcoverro@anicura.es; 3 Department of Neurology and Neurosurgery, AniCura Ars Veterinaria Hospital Veterinari, 08034 Barcelona, Spain; 4 Department of Neurology and Neurosurgery, Anderson Moores Veterinary Specialists, Winchester SO21 2LL, UK; edward.ives@andersonmoores.com

**Keywords:** canine, disc extrusion, involuntary movement, jerk, movement disorder, MRI, neck pain, “shock-like movement”, spinal pain, twitching

## Abstract

Cervical myoclonus (CM) has been associated with intervertebral disc extrusion (IVDE), with a higher prevalence in French Bulldogs. The presence of CM in other breeds and with other aetiologies has not been reported. The purpose of this study was to describe the signalment, neurological examination, neuroanatomical localisation and grade, imaging findings, diagnosis, treatment, follow-up and resolution of CM in dogs. An observational multicentred retrospective analysis identified 173 dogs with CM; of those, 113 met the inclusion criteria. French Bulldogs (n = 52/113, 46%), Beagles (n = 8/113, 7.1%), Chihuahuas and Shih-Tzus (n = 6/113 for each, 5.31%) were the most affected breeds. Apparent cervical pain was the most common finding on neurologic examination (n = 70/113, 62%). Magnetic resonance imaging (MRI) was consistent with nerve root impingement in 17% (n = 19/113) of the dogs. The most frequently diagnosed conditions were degenerative (n = 100/113, 88.5%), inflammatory (n = 8/113, 7.1%), neoplastic (n = 3/113, 2.7%), vascular (n = 1/113, 0.9%) and congenital (n = 1/113, 0.9%) in origin. Dogs with a neoplastic aetiology tended to be older than those with other causes. Follow-up was recorded in 77 dogs, and 75 of these (n = 75/77, 97.4%) had resolution of the CM. The results supported that cervical myoclonus can be caused by various underlying conditions and can affect different dog breeds.

## Simple Summary

Neck spams have been associated with acute intervertebral disc herniation (IVDH) in dogs, with a higher prevalence in French Bulldogs. It is unknown if neck spasms occur in other breeds with other diseases. This multicentred study aimed to describe neck spasms in a large number of dogs, including its distribution regarding signalment, neurological signs, magnetic resonance imaging (MRI) findings, suspected diagnosis, treatment and outcome. A total of 113 dogs with neck spasms as part of their presenting clinical signs were included in the study. The French Bulldog (n = 52/113), Beagle (n = 8/113), Chihuahua (n = 6/113) and Shih-Tzu (n = 6/113) were the most frequently affected breeds. Neck pain as a single finding was the most common abnormality in the neurological examination. The most common diagnosis was acute IVDH, followed by inflammatory, neoplastic, congenital and vascular diseases. The median age of dogs with neoplastic lesions (134 months) was higher than those of dogs with inflammatory (96 months), degenerative (72 months), vascular (24 months) and congenital (21 months) conditions. Follow-up was recorded in 77 dogs (68%), and 75 of those had resolution of the neck spasms at that time. The results supported that neck spasms can be caused by various underlying conditions and can affect different dog breeds.

## 1. Introduction

Myoclonus is the term used to describe a sudden, brief, shock-like contractions affecting one or more body regions [1,2,3,4,5,6]. It can originate from various anatomical levels, including cortical, cortical-subcortical, subcortical-nonsegmental, segmental, and peripheral sources [7]. Differentiating myoclonus from other involuntary movement disorders remains a significant diagnostic challenge in veterinary neurology. Accurate identification of its origin often necessitates the combined use of electroencephalography (EEG) and electromyography (EMG) to determine the neuroanatomical localisation and to distinguish between epileptic and non-epileptic forms.

Spinal myoclonus is a form of myoclonus that originates either from the subcortical-nonsegmental or segmental levels of the spinal cord, typically involving one or several adjacent spinal segments. Spinal segmental myoclonus (SSM) arises from disinhibition of spinal interneurons, resulting in hyperexcitability of ventral horn motor neurons [8]. Cervical myoclonus (CM) represents a subtype of spinal myoclonus specifically affecting the cervical region. A wide range of aetiologies have been associated with spinal myoclonus in humans, including idiopathic as well as secondary causes, such as trauma, spinal cord injury, demyelinating diseases, infections, adverse drug reactions, and IVDH following spinal surgery, intramedullary and extramedullary space-occupying lesions, vascular abnormalities, degenerative conditions, cervical spondylosis, spinal anaesthesia blocks, electrical injury, and glial scarring [9,10,11,12].

In veterinary medicine, myoclonus is broadly categorised as either epileptic or non-epileptic [3,13], with each type linked to distinct underlying aetiologies [3,7,14]. Epileptic myoclonus encompasses conditions such as Lafora disease, neuronal ceroid lipofuscinosis, juvenile myoclonic epilepsy, and idiopathic myoclonic epilepsy [3]. Non-epileptic myoclonus includes cases associated with canine distemper virus infection, startle disease, and hemifacial spasm [3], as well as cervical syringomyelia (SM) [15], cervical IVDH [1,16], hypercalcemia [17], and the subarachnoid administration of morphine [18,19]. Cervical myoclonus has been documented in a recent study investigating the prevalence of cervical “jerks” in dogs undergoing computed tomography (CT) for cervical pain or suspected cervical myelopathy. The findings indicated that cervical jerks are an uncommon clinical sign and appear to be exclusively associated with IVDE, with French Bulldogs making up 65% of the 20 affected cases [1]. In another report, CM was described as a consequence of nerve root compression secondary to intervertebral disc disease (IVDD) in the cervical region [16].

Cervical pain is a common yet non-specific clinical and neurological finding in dogs. The underlying mechanisms have been broadly categorised into three groups: mechanical, neuropathic and referred [20]. Mechanical causes originate from spinal structures such as vertebral ligaments, musculature, intervertebral discs (IVD), facet joint capsules, dorsal root ganglia, vertebral periosteum and meninges. Neuropathic pain arises from irritation, either mechanical or chemical, of nerve roots. Additionally, cervical pain may occur secondarily due to conditions not originating in the cervical region, such as intracranial diseases, and be classified as referred [20]. A recent study supported CM as a clinical sign associated with cervical pain [1].

The main aim of this study was to broaden the understanding of CM clinical distribution. Thus, the objectives of this study were to describe the signalment, neurological examination findings, neuroanatomical localisation, presence of cervical pain, MRI findings, final or presumptive diagnosis, treatment and clinical outcome in a large sample of dogs with CM that were evaluated at the neurology departments of three referral hospitals in the United Kingdom (UK). Additionally, we aimed to assess whether specific features of the signalment or neurological examination were particularly common with underlying causes of CM. We hypothesised that conditions other than IVDH can also cause CM, and that breeds other than French Bulldogs may also be affected. Furthermore, we hypothesised that cervical pain is a highly prevalent clinical sign in dogs presenting with CM.

## 2. Materials and Methods

The present investigation was designed as a multicentre, retrospective observational study. Ethical approval was obtained from the Ethical Review Board of the Department of Veterinary Medicine, University of Cambridge (CR880).

### 2.1. Case Identification

The medical databases of the Neurology Services at three referral hospitals were retrospectively reviewed for the period between January 2014 and December 2023 to identify dogs with a history or clinical finding at presentation of CM using the following keywords: “cervical myoclonus”, “cervical twitching” and “cervical spasms” or “jerks”.

Dogs were included if they met the following inclusion criteria: (1) CM was either directly observed, documented via video footage, or appropriately described by the referring veterinarian or owner; and (2) magnetic resonance imaging (MRI) of the cervical vertebral column was performed. Cervical myoclonus was defined as a brief, abrupt and involuntary contraction of muscle groups in the cervical region, without apparent loss of consciousness and without involvement of other body parts. An appropriate description of CM by owners or referring vets included the words “twitches”, “jerks”, “electric shock-like movement”, or “spasms” involving the cervical region. Cases were excluded if any of the following applied: (1) medical records or MRI of the cervical region were incomplete or unavailable; (2) myoclonus involved muscle groups other than those of the cervical region; (3) the lesion was localised to regions of the nervous system other than the C1-T2 spinal segment; (4) dogs had a history or neurological findings suggestive of intracranial disease or seizures; (5) concurrent presence of two or more distinct diseases; (6) diagnosis of a non-neurological condition (e.g., orthopaedic or dermatological); or (7) no final diagnosis could be established. Cases were included even if MRI findings showed coexisting conditions such a Chiari-like malformation, mild intervertebral disc protrusions (IVDP), in situ intervertebral disc degeneration, secondary intramedullary changes (e.g., gliosis, oedema), or the presence of an atlantoaxial band in combination with the primary cervical spinal lesion diagnosed as the cause for CM.

CM-dogs were not associated with sleep, were not induced by external stimuli, and were not stimulus-sensitive. CM episodes occurred while the dog was standing, sitting or lying in a sternal recumbency, and multiple episodes were reported in each case.

While the observed cervical movements can overlap other muscle fasciculations or muscle twitches, most of the movement presented by the included dogs is myoclonus. Fasciculations are defined as spontaneous, involuntary contractions and relaxations of small groups of muscle fibres, typically presenting as subtle, localised twitches that do not result in overt movement of a limb or body part [13,21]. In contrast, the included myoclonic movements differ from fasciculation by showing greater amplitude and leading to visible displacement of the neck in a shock-like fashion [1,2,3,4,5,6].

Signalment, medical history, onset and progression of clinical signs, presence of pain, MRI, surgical findings and other clinicopathological data were used to establish a final or presumptive diagnosis. Follow-up information was obtained through review of the medical records.

### 2.2. Data Collection

The medical records were reviewed for the following variables: (1) age, (2) sex, (3) breed, (4) neuroanatomical localisation, (5) neurological grade, (6) presence of apparent cervical pain at presentation, (7) disease category, (8) final or presumptive diagnosis, (9) MRI findings, (10) lesion localisation on MRI, (11) type of treatment, (12) surgical technique (if applicable), (13) follow-up status, (14) follow-up duration and (15) resolution of CM during follow-up.

Age was categorised into three groups: (<24 months, 24–96 months, >96 months); sex was recorded as one of four categories: (female entire, female neutered, male entire, male neutered); and neuroanatomical localisation was classified as a C1-C5, C6-T2, or a C1-T2 spinal segment. Disease category was determined using the DAMNITV mnemonic.

Neurological grade was classified into three categories (1, 2 or 3) based on the neurological examination: (1) cervical pain without other neurological deficits; (2) ambulatory tetraparesis with proprioceptive deficits, with or without cervical pain; and (3) non-ambulatory tetraparesis with proprioceptive deficits, with or without cervical pain [22]. These are summarised in Table 1.

Cervical pain was assessed at presentation (yes/no) and was also noted when it was the sole finding on neurological examination.

Magnetic resonance imaging features of the cervical region included the following: nerve root compression (yes/no); epidural haemorrhage (yes/no); contrast enhancement of the meninges (yes/no); and contrast enhancement of the nerve roots (yes/no). Lesion localisation based on MRI finding was recorded relative to the affected IVD space, spinal cord segment or broader neuroanatomical region (C1-C5, C6-T2, C1-T2).

Treatment was categorised as medical, surgical or euthanasia. When surgical intervention was performed, the technique was recorded (ventral slot, cervical hemilaminectomy, cervical dorsal laminectomy or other). Follow-up information included whether follow-up was available (yes/no), the duration of follow-up when available (measured in days from diagnosis, categories being ≤7 days and >7 days), and whether CM had resolved by that time (yes/no). Follow-up was conducted either in person by the referring veterinarian or veterinary neurologist, or by telephonic survey. Resolution of CM was defined as the absence of observable myoclonic episodes, as reported by the owners or confirmed by a veterinarian during clinical reassessment.

All cases were managed either by a board-certified veterinary neurologist or by a veterinary neurology resident under supervision. All medical records and MRI studies were reviewed by a board-certified veterinary diagnostic imager or neurologist (L.A.) or by a veterinary neurology resident (A.M.). All surgical procedures were performed by a European College of Veterinary Neurology (ECVN) resident under the supervision of an ECVN-boarded specialist, or by an ECVN-boarded specialist.

### 2.3. Diagnostic Imaging

Dogs were anaesthetised and positioned in lateral, dorsal or sternal recumbency. Acquisition of MR images was performed using either a 0.27-Tesla (Esaote VetMR Grande, Genova, Italy) or 1.5-Tesla MRI scanner (Siemens Magnetom Essenza™—Siemens Shenzhen Magnetic Resonance Ltd., Shenzhen, China; Siemens AG, Erlangen, Germany). Required sequences included at minimum sagittal and transverse T2-weighted (T2W) images and transverse T1-weighted (T1W) images. Additional sequences, such as fluid-attenuated inversion recovery (FLAIR) and post-contrast T1W, were reviewed when available but were not mandatory for inclusion. The cervical vertebral column was imaged in all dogs based on neurological examination findings. All acquired sequences and imaging planes acquired were made available to the authors for internal review.

The MRI-based presumptive diagnosis was determined by combining imaging findings with clinical data including signalment, medical history, onset, progression and presence of pain. For cases diagnosed with IVDH, the following imaging features were assessed: nerve root impingement (yes/no), epidural haemorrhage (yes/no), contrast enhancement of the meninges/nerve roots (yes/no) and nerve root enlargement (yes/no). Nerve root enlargement and/or contrast enhancement in the absence of significant spinal cord compression was considered consistent with neuritis.

### 2.4. Statistical Analysis

Descriptive statistical methods were applied to evaluate the collected data. For continuous variables with normal distribution, results were expressed as mean ± standard deviation (SD), whereas non-normally distributed data were summarised using the median and interquartile range (IQR). Categorical variables were reported as frequencies and percentages. All statistical analyses were conducted using the VassarStats online platform.

## 3. Results

**Figure 1:**
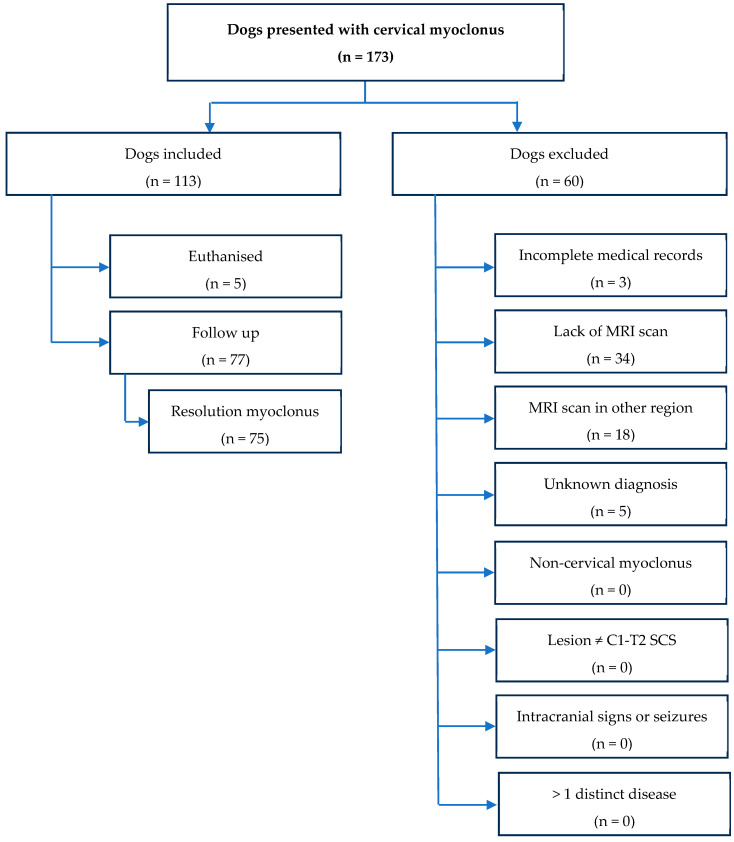
Flow chart demonstrating data collection.

A total of 173 dogs with CM were initially evaluated. Of these 173 dogs, a total of 60 dogs (60/173, 34.7%) were excluded for the following reasons: (1) absence of MRI study in 34 dogs (34/60, 57%); (2) MRI performed in two different anatomical regions in 18 dogs (18/60, 30%); (3) lack of a definitive or presumptive diagnosis in 5 cases (5/60, 8%); and (4) incomplete clinical history in 3 cases (3/60, 5%). Consequently, 113 dogs (113/173, 65.3%) met the inclusion criteria and were included in the analysis as CM-dogs. The inclusion/exclusion process is summarised in Figure 1.

### 3.1. Signalment

Of the 113 CM-dogs included, 7 out of 113 (7/113) were younger than 24 months, 70/113 (62%) were between 24 and 96 months and 36/113 (31.8%) were older than 96 months. The median age at the time of MRI was 72 months (range, 48–106 months). Regarding sex distribution, 12 CM-dogs (12/113, 10.6%) were female entire, 41/113 (36.3%) were female neutered, 13/113 (11.5%) were male entire and 47/113 (41.6%) were male neutered.

Among the 22 dog breeds represented in the study, the French Bulldog was the most common (52/113, 46%), followed by the Beagle (8/113, 7.07%), Chihuahua (6/113, 5.31%) and Shih-Tzu (6/113, 5.31%). Other observed breeds were Cavalier King Charles Spaniel (5/113, 4.42%), Cocker Spaniel (5/113, 4.42%), Dachshund (5/113, 4.42%), Jack Russell Terrier (4/113, 3.54%), Springer Spaniel (4/113, 3.54%), crossbreed (3/113, 2.65%), Cockerpoo (3/113, 2.65%), Labrador (2/113, 1.77%) and one of each of the following 10 breeds (1/113, 0.88%): American Bulldog, Bichon Frisé, Boston Terrier, Coton de Tulear, Dalmatian, Irish Terrier, Lagotto Romagnolo, Miniature Schnauzer Terrier, Patterdale and Whippet. The main findings are summarised in Table 2.

### 3.2. Findings in the Neurological Examination

The most common clinical finding was cervical pain without neurological deficits, observed in 70/113 (62%) dogs, followed by neurological deficits localised to the C1-C5 spinal segment in 32/113 (28.3%) dogs, to the C1-T2 spinal segment in 7/113 (6.2%) dogs, and to the C6-T2 spinal segment in 4/113 (3.5%) dogs, with or without cervical pain. The majority of CM-dogs (108/113, 95.6%) had cervical pain identified during neurological examination. Among these 108 CM-dogs, 70 (70/108 (64.8%) exhibited cervical pain as the sole finding, 29 (29/108, 26.9%) had neurological deficits localised to the C1-C5 spinal segment, 5 (5/108, 4.6%) to the C1-T2 spinal segment, and 4 (4/108, 3.7%) to the C6-T2 spinal segment.

According to the neurological grade classification, the majority of cases were classified as grade 1, presenting with cervical pain only (70/113, 62%), followed by grade 2 (38/113, 33.6%) and grade 3 (5/11, 4.4%). Among grade 2 CM-dogs, 30 (30/38, 79%) also exhibited cervical pain. All grade 3 CM-dogs (5/5, 100%) had concurrent cervical pain identified on examination. Clinical data are summarised in Table 3.

### 3.3. MRI Characteristics

Among the 113 CM-dogs included in the study, nerve root impingement was suspected in 17% of the cases (19/113), all of which were associated with IVDE (19/88, 21.6%). Three cases of nerve root impingement had nerve root enlargement (3/19, 15.8%), of which 2 (2/3, 66.7%) also showed contrast enhancement.

Overall, contrast enhancement was identified in 10.6% of the cases (12/113). The enhanced structures included nerve roots (6/12, 50%), meninges (3/12, 25%), spinal cord (1/12, 8.3%), extradural material associated with IVDH (1/12, 8.3%, 95% CI 1.5–35.4) and dorsal root ganglion (1/12, 8.3%).

Eight CM-dogs (8/113, 7.1%) showed nerve root enlargement, with strong contrast enhancement observed in 4/8 (50%). Extradural haemorrhage associated with IVDE was suspected in 3/113 (2.7%) cases, all of which were surgically confirmed.

Among the 70 CM-dogs that had a normal neurological examination with cervical pain as the sole clinical finding (70/113, 62%), MRI revealed a C1-C5 lesion in 88.6% (62/70) of the cases, a C1-T2 lesion in 8.6% (6/70) of the cases, and a C6-T2 in 2.9% (2/70) of the cases. All CM-dogs with a neuroanatomical localisation to the C1-T2 spinal segment had lesions affecting the C1-C5 spinal segment. Among all the cases included in the study, only 4 CM-dogs (4/113, 3.5%) had a lesion exclusively affecting the C6-T2 spinal segment, and all of these were initially localised differently on the neurological examination, either to the C1-C5 spinal segment (2/4, 50%) or with cervical pain only (2/4, 50%).

### 3.4. Diagnosis

Of those CM-dogs included in the study, 100 CM-dogs (100/113, 88.5%) were diagnosed with suspected degenerative diseases. Among these, 88% had IVDE (88/100), 5% had IVDP (5/100), 3% had multiple IVDPs (3/100), 2% had hydrated nucleus pulposus extrusion (HNPE) (2/100) and 2% had SM (2/100).

Out of 113 cases, 8 (8/113, 7.1%) were diagnosed with suspected inflammatory disease, including 3/8 (37.5%) with myelitis/meningomyelitis of unknown origin (MUO), 2/8 (25%) with steroid responsive meningitis arteritis (SRMA), 2/8 (25%) with neuritis and 1/8 (12.5%) with idiopathic bilateral C2 hypertrophic ganglioneuritis.

A total of three cases (3/113, 2.7%) were diagnosed with neoplastic lesions. Among these, 2 CM-dogs (66.7%) had suspected intramedullary neoplasia, and 1 CM-dog (33.3%) had a suspected osteosarcoma without evidence of spinal invasion.

One case was classified as anomalous disease with atlantoaxial instability (AAI) (1/113, 0.9%) and another case was classified as vascular disease, with a suspected fibrocartilaginous embolic myelopathy (FCEM) (1/113, 0.9%). Conditions found in dogs with CM are summarised in Table 4.

CM-dogs with neoplastic lesions were older ($\bar{x}$ 134 months, IQR: 97–212) compared to those with inflammatory ($\bar{x}$ 96 months, IQR 38–134) or degenerative diseases ($\bar{x}$ 72 months, IQR: 48–106). CM-dogs with IVDPs or SM were older ($\bar{x}$ 132 months, IQR: 88–165) than those with IVDE ($\bar{x}$ 70 months, IQR: 42–97).

All CM-dogs diagnosed with inflammatory diseases and two (2/3, 66.7%) with neoplastic diseases exhibited cervical pain on examination. The majority of dogs with degenerative diseases (97/100, 97%) also had cervical pain.

The most common extruded IVD in CM-dogs was C3-C4, identified in 46.6% (41/88), followed by the C2-C3 in 31.8% (28/88), the C4-C5 in 14.8% (13/88) and both C5-C6 and C6-C7 in 3.4% of the cases (3/88).

The French Bulldog was the most commonly affected breed with IVDD, accounting for 50/88 dogs (57%), followed by Beagles in 8/88 (9.1%) and Shih-Tzus in 5/88 (5.7%). The majority of French Bulldogs (51/52, 98.1%) were diagnosed with degenerative conditions, with 50/52 (96.2%) having IVDE and 1/52 (1.9%) diagnosed with IVDP. One (1/52, 1.9%) French Bulldog was diagnosed with idiopathic bilateral C2 hypertrophic ganglioneuritis.

### 3.5. Treatment

Of the 88/113 (77.9%) cases diagnosed with IVDE, 65/88 (73.9%) were initially treated surgically, 21/88 (23.9%) were treated medically and 2/88 (2.3%) were euthanised. Overall, 70/88 (80%) dogs underwent spinal surgery: 65/70 (93%) had a ventral slot procedure and 5/70 (7%) underwent cervical hemilaminectomy. Medical treatment consisted of rest, along with a combination of oral non-steroidal anti-inflammatory (NSAIDs) drugs, gabapentin (8–15 mg/kg PO TID), paracetamol (10–15 mg/kg IV or PO TID) and methadone (0.1–0.3 mg/kg IV q4h) for analgesia.

Only non-infectious inflammatory conditions were reported (8/113, 7.1%), and all were managed medically with immunosuppressive therapy. Treatment consisted of gradually tapering prednisolone (starting dose 2 mg/kg BID PO). Cases presenting with severe neurological signs also received cytosine arabinoside (200 mg/m^2^ over 8–24 h, SC or IV) and/or cyclosporine (5–10 mg/kg BID PO).

All neoplastic cases (3/113, 2.7%) were managed with palliative treatment, which included glucocorticoids (0.5 mg/kg PO SID) and analgesia with gabapentin (8–15 mg/kg PO TID) and/or paracetamol (10–15 mg/kg IV or PO).

The only case diagnosed with AAI (1/113, 0.9%) was initially managed medically with rest and a combination of NSAIDs, gabapentin (8–15 mg/kg PO TID) and paracetamol (10–15 mg/kg PO TID); however, due to a lack of clinical improvement with the medical treatment, ventral surgical stabilisation was subsequently performed (transarticular screws and polymethylmethacrylate—PMMA).

One case (1/113, 0.9%) diagnosed with FCEM was managed medically and with rehabilitation, which included a combination of physiotherapy and hydrotherapy, along with oral NSAIDs and paracetamol (10–15 mg/kg PO TID) for analgesia.

### 3.6. Follow-Up and Outcome

Seventy-seven CM-dogs (77/113, 68.1%) had follow-up data available, ranging from 5 to 365 days, with a mean follow-up period of 7 weeks from the day of diagnosis. These included 69/77 with degenerative, 7/77 inflammatory and 1/77 with anomalous conditions. Overall, 97.4% of the CM-dogs (75/77) showed resolution of CM.

Of the two CM-dogs in which CM persisted, one was a 2-year-old Bichon Frise with neurological grade 3 and localised C1-C5 spinal segment, diagnosed with a suspected meningomyelitis. This dog deteriorated and was euthanised. The other was a 15-year-old Cavalier King Charles with neurological grade 1 and localised with cervical hyperesthesia, diagnosed with suspected SM. Although the CM did not completely resolve in this case, clinical signs, including CM, cervical pain and gait abnormalities, improved 7 weeks after diagnosis with medical treatment consisting of 0.4 mg/kg of memantine SID PO, 3.4 mg/kg of pregabalin BID PO and 1.3 mg/kg of robenacoxib SID PO.

In one dog initially treated surgically (1/65, 1.5%), CM did not resolve following surgery. This CM-dog was subsequently treated with an ultrasound-guided paravertebral perineural glucocorticoid injection, which resulted in subsidence of CM.

Five of the dogs that were initially diagnosed with IVDE and managed medically (5/21, 23.8%) failed to improve and subsequently underwent surgical intervention, achieving complete resolution of CM.

Of the 75 dogs with resolution of CM, 45 (45/75, 60%) had a neurological grade 1, 26/75 (34.7%) had a grade 2 and 4/75 (5.3%) had a grade 3 at presentation. Of the 88 dogs diagnosed with IVDE, follow-up was available for 59/88 (67%), and all of them (59/59, 100%) experienced resolution of clinical signs. Follow-up and resolution are summarised in Table 5.

## 4. Discussion

Cervical myoclonus is a form of involuntary movement that has been reported in association with IVDE, particularly in the French Bulldog [1]. Previous studies have also suggested that CM is associated with cervical pain in dogs with IVDH [1,16]. In this multicentred group study of 113 dogs, CM was observed in dogs with a range of underlying aetiologies and across different dog breeds, indicating a lack of clinical specificity.

Cervical pain emerged as the most common clinical finding, with only 4% of the affected dogs perceived as non-painful.

All dogs included in the study were required to have a diagnosis of CM in conjunction with an identifiable underlying disease, affecting the C1-T2 spinal segment and/or their associated nerve roots. The most frequently observed categories of disease were degenerative, inflammatory and neoplastic disorders. Less commonly, anomalous and vascular aetiologies were also identified. This distribution of diseases with CM has not been previously documented in the veterinary literature.

Cervical myoclonus in dogs has previously been reported only in association with IVDE [1,16]. In the present study, additional degenerative conditions were identified, including IVDP, HNPE and SM. Prior studies reported CM in association with IVD lesions localised between the C2 and C5 vertebrae [1,16]. However, our larger sample demonstrated that CM can occur, albeit infrequently, with IVDE of the C5-C6 and C6-C7 IVD, as well as in one case of AAI. These findings suggest that CM can be observed with lesions spanning the entire cervical vertebral column.

The French Bulldog has been reported as the most commonly affected breed presenting with CM secondary to IVDE [1]. The findings of the present study are consistent with this observation; however, one potential confounding factor is that the French Bulldog is the second most referred breed to veterinary hospitals in the UK [23]. Additionally, IVDE is the most prevalent neurological disorder in this breed, with approximately 40% of cases involving the cervical spine [9,24]. Further research is warranted to determine whether French Bulldogs possess a specific biological predisposition for CM. This study also identified other affected breeds, including Beagles, which are similarly prone to cervical IVDE, as well as Chihuahuas and Shih-Tzus [25].

Inflammatory conditions represented the second most common disease category in the CM-dogs, but still only accounted for 7.1% (8/113) of cases. The most frequently diagnosed inflammatory disorders were meningomyelitis of unknown origin (MUO), with no cases of infectious inflammatory disease identified in this sample population. This finding is consistent with previous literature indicating that non-infectious inflammatory conditions are significantly more prevalent than infectious aetiologies, with SRMA and MUO representing approximately 79% of all inflammatory spinal cord disorders in dogs [26]. To date, CM without the involvement of other body parts has not been reported in dogs with inflammatory conditions, making this finding novel.

As expected, CM-dogs diagnosed with inflammatory conditions tended to be younger, with those affected by SRMA being the youngest ($\bar{x}$ 11.5 months), followed by those with suspected MUO ($\bar{x}$ 51 months) [26,27]. All the cases of suspected MUO occurred in toy- or small-breed dogs, consistent with established breed predisposition for this condition [28].

Neoplastic conditions were infrequently identified, representing 2.7% (3/113) of our CM-dog sample. Among these, one case had neoplasia of the surrounding non-neurological cervical structures, while the remaining two presented with intramedullary spinal cord tumours. As expected, neoplasia was diagnosed in older dogs ($\bar{x}$ 134 months). Cervical neoplasia is generally rare in dogs and, when present, is less commonly associated with cervical pain [29].

Anomalous and vascular conditions were identified as the sole underlying cause of CM in two dogs (1.8%; 2/113), one diagnosed with AAI and the other with FCEM. Both dogs exhibited cervical pain on palpation. This finding aligns with prior reports in which cervical pain was the most common clinical sign associated with AAI [30,31]; however, CM has not been previously described in that context. Although FCEM is considered an uncommon cause of cervical myelopathy and pain, one study reported its presence in 30 of 478 dogs evaluated for cervical spinal cord disease, with 12 of those cases exhibiting apparent cervical pain [32]. Again, CM was not reported in that cohort. As with inflammatory conditions, neoplastic, vascular, and anomalous findings have not previously been reported with CM, making this a novel observation.

Overall, the distribution of the aetiologies observed in this study is consistent with the reported prevalence of aetiologies for canine cervical myelopathies [33]. It is possible that CM may not be linked to a specific underlying disease but rather may share common pathophysiological pathways with pain perception or result from lesions affecting currently unidentified anatomical structures, irrespective of cause. In support of this, MRI analysis in this study did not reveal a consistent pattern of structural or anatomical abnormalities among CM-dogs, nor was there an overrepresentation of features such as haemorrhage or contrast enhancement.

In contrast, CM has been frequently reported in human medicine, where it is associated with a broad spectrum of aetiologies, including infectious and inflammatory/immune-mediated myelopathies, spinal cord neoplasia, SM, spinal cord trauma, vascular abnormalities, degenerative motor neuron disease, demyelination, cervical spondylosis, and IVDH [5,9,34]. The range of conditions identified in our canine group closely parallels these findings, highlighting potentially shared pathophysiological mechanisms.

Cervical pain was the predominant clinical finding on neurological examination, observed in 95.6% (108/113) of the CM-dogs. Among these, 65% (70/108) did not exhibit any neurological deficits to suggest spinal cord dysfunction. This supports previous findings suggesting that CM may reflect cervical pain in the absence of myelopathic signs in up to 75% of the cases [1]. However, further studies are warranted to characterise the association, if any, between CM and pain.

The most frequently identified underlying conditions in this study were IVDE (78%) and inflammatory disorders (7.1%), including SRMA, MUO, neuritis, and C2 ganglioneuritis. In support of these findings, cervical pain has been reported as the most common clinical sign associated with cervical IVDE, with myelopathic deficits less frequently observed [1,35,36]. Furthermore, a separate study identified IVDE as the second most likely diagnosis, following SRMA, in dogs presenting with cervical pain alone, without neurological deficits [37]. Similarly, spinal pain is a well-recognised clinical feature of inflammatory conditions [28]. However, myoclonus involving only the cervical muscles has only recently been reported in dogs with IVDE [1].

Cervical pain may arise from mechanical causes (e.g., lesions involving the cervical vertebral column or supporting structures), neuropathic mechanisms (e.g., lesions of the somatosensory system involving the spinal cord, dorsal root ganglia, or spinal nerves), or from lesions outside the cervical region in cases of referred pain [20]. Myoclonus may also be generated by lesions that alter the microenvironment of the spinal cord, disrupting the function of inhibitory interneurons involved in its generation, or by lesions causing hyperexcitability of peripheral nervous structures, which could contribute to the development of myoclonic activity [8]. In our sample, CM was not further classified as uni- or bilateral nor was it categorised based on the distribution of affected muscle groups, and pain was not assessed quantitatively; these are details that could have offered additional insight into the origin of myoclonus. Further studies employing well-characterised descriptions of myoclonus are warranted. Such studies may help elucidate whether CM originates from subcortical or peripheral mechanisms [38,39,40].

The study identified five CM-dogs that did not exhibit signs of cervical pain. Interestingly, four of these cases were diagnosed with IVDH, including two with IVDP and two with IVDE. The fifth case involved a neoplastic condition. Given that pain assessment in this sample was qualitative in nature, the absence of overt pain behaviours does not definitively confirm that these animals were pain-free.

The nerve root signature is a neurological sign generally interpreted as indicative of pain [41]. The presence of this sign was not recorded or analysed in this study, and it remains unclear why cervical IVDH can, in some cases, presented with CM and in others with the nerve root signature of the thoracic limbs. The authors also hypothesise whether different pathomechanisms or types of pain underlie these two distinct clinical signs.

Cervical transforaminal epidural glucocorticoid injections are commonly used in human medicine for the management of radicular pain [42,43]. In canine veterinary medicine, perineural glucocorticoid injections in the cervical region have been reported to provide effective analgesia in dogs with cervical foraminal IVDH [44,45], however these cases did not have reported CM. In the present study, a similar approach was used in one dog with CM and nerve root impingement due to IVDE. This CM-dog did not respond to initial surgical intervention but experienced complete resolution of CM following a perineural glucocorticoid injection, suggesting a potential role for targeted anti-inflammatory therapies in selected cases.

Cervical myoclonus in dogs appears to carry a favourable prognosis, largely dependent on the underlying aetiology. In the present sample, 97.4% (75/77) of CM-dogs with follow-up experienced resolution of CM following treatment directed at the primary disease. Notably, in five CM-dogs with IVDH, the CM did not initially respond to medical management but subsequently responded to surgical intervention. These findings are consistent with prior reports in both human [9,10] and canine veterinary literature [1], which have described resolution of CM following surgical treatment of IVDH.

Overall, the prognosis for CM in this sample was excellent, with full resolution achieved in 75 of 77 CM-dogs for which follow-up data were available. Prognosis was particularly favourable in cases with IVDH, consistent with previous findings [1]. These results emphasise the importance of identifying and addressing the underlying aetiology in CM-dogs, as appropriate treatment often leads to complete clinical recovery.

In human medicine, adjunctive therapies such as cervical spinal cord stimulation have been employed to manage CM-plus associated with degenerative compressive myelopathies. The proposed mechanisms include the modulation of neurotransmitter release, the activation of cerebral regions involved in pain inhibition, the influence on spinal interneurons or motor neurons via transsynaptic pathways, and the activation of monosynaptic reflex arcs [46]. However, cervical spinal myoclonus secondary to SM appears more refractory to treatment, with some cases remaining static over a two-year period [47]. Conversely, other reports have documented the resolution of CM following surgical intervention, such as Chiari decompression and duraplasty in patients with SM associated with Chiari malformation [48]. In the present study, two dogs were diagnosed with CM and SM. One case experienced complete resolution of CM, while the other showed clinical improvement following medical management. These findings support the notion that CM with SM may exhibit variable treatment responses, potentially influenced by the underlying micropathology and the therapeutic approach employed.

Several limitations arise from the retrospective and multicentre design of this study. The classification of CM was based on direct clinical observation and review of home-recorded videos, without the benefit of EEG or EMG. Based on the inclusion and exclusion criteria, along with the clinical characteristics of the myoclonus, a nonepileptic origin of CM was suspected in all cases. Importantly, resolution of CM occurred in all dogs for which the underlying cervical pathology was successfully treated, further supporting a non-epileptic aetiology. Nevertheless, in the absence of EEG data, the possibility of epileptic myoclonus, though considered unlikely, cannot be definitively excluded.

Additionally, it is possible that some dogs included in the study may have exhibited other forms of intermittent movement disorders, such as tremors or fasciculations that were misclassified as CM. This highlights the need for future studies employing standardised video analysis protocols and electrophysiological testing to improve diagnostic accuracy.

Follow-up information was not available for all dogs, and follow-up protocols were not standardised across the participating institutions. The average follow-up duration was approximately seven weeks, and long-term follow-up was lacking in the majority of cases. Despite these limitations, CM resolved in all but two cases during the short-term follow-up, suggesting that CM may be a transient clinical sign that responds rapidly to appropriate treatment of the underlying condition. However, the possibility of recurrence beyond the documented follow-up period cannot be excluded and unanticipated changes in neurological status may have gone undetected.

Pain assessment in this study was qualitative in nature, which may have limited our ability to detect cervical discomfort in the 5 CM-dogs classified as pain-free. Additionally, we were unable to identify any consistent altered anatomical structures or MRI features in the CM-dogs. The study utilised two different magnetic field strengths, which may introduce variability in image quality and resolution. The MRI protocols were not standardised across institutions, although all dogs underwent orthogonal T2w and T1w sequences covering the cervical spine. Furthermore, CSF analysis or post-contrast MRI were not performed in all dogs, raising the possibility that additional mechanisms or anatomical features contributing to CM may have been missed. None of the CM-dogs in this study underwent histopathological confirmation of diagnosis, limiting the ability to definitively correlate CM with underlying pathology.

The present study broadened the characterisation of CM in dogs with cervical spinal cord diseases. The clinical significance of CM in the diseases reported herein remains uncertain. Cervical myoclonus appears to be a clinical sign that may accompany a variety of cervical spinal conditions and notably, tends to resolve following appropriate treatment of the underlying disease. Most CM-dogs exhibited concurrent cervical pain without overt myelopathic deficits. However, the presence of CM in dogs lacks specificity with respect to underlying pathology, demographic factors, or prognostic value. While the potential link between CM and pain is a plausible hypothesis, further association studies with electrophysiological investigations and standardised imaging protocols are required to clarify its pathophysiological basis and clinical relevance. Additionally, large epidemiological studies can give better insight on the prevalence of CM and clarify whether CM can exist as its own entity.

## 5. Conclusions

In conclusion, CM in dogs can occur with a wide range of underlying conditions, most commonly IVDH, followed by inflammatory and neoplastic diseases. Notably, the majority of affected dogs also exhibited cervical pain, irrespective of the underlying aetiology. In nearly all cases, CM resolved following treatment of the primary disease, reinforcing that it is a secondary clinical sign rather than a distinct pathological entity.

Cervical myoclonus was most frequently identified in French Bulldogs, often in the context of cervical IVDE. Once the underlying disease is addressed, the CM resolves with an excellent prognosis. Further epidemiological and prospective, standardised studies are needed to elucidate the pathophysiological mechanisms of CM, particularly its potential link to pain and its diagnostic and prognostic relevance in canine neurology.

## Figures and Tables

**Table 1:** Neurological grade classification.

Neurological Grade	Neurological Status
1	Cervical pain
2	Ambulatory with proprioceptive deficits +/− cervical pain
3	Non-ambulatory with proprioceptive deficits +/− cervical pain

**Table 2:** Demographic data.

Signalment	Variable
Most common breeds	French Bulldog (n = 52/113)
	Beagle (n = 8/113)
	Chihuahua (n = 6/113)
	Shih-Tzu (n = 6/113)
Sex	Female, n = 53/113 (41 neutered)
	Male, n = 60/113 (47 neutered)
Age	Median = 72 months

**Table 3:** Categorical data for presence of cervical pain, neuroanatomical localisation and neurological grade.

**Cervical Pain**	Yes	n = 108/113
	No	n = 5/113
**Neuroanatomical localisation**	C1-C5 spinal segment	n = 32/113
	C6-T2 spinal segment	n = 4/113
	C1-T2 spinal segment	n = 7/113
	Only cervical pain	n = 70/113
**Neurological grade**	1	n = 70/113
	2	n = 38/113
	3	n = 5/113

**Table 4:** Conditions found in dogs with cervical myoclonus.

Disease Category (DAMNITV ^1^)	Diseases	Total Cases n = 113	Pain (Yes)
Degenerative	Intervertebral disc disease (IVDD)	98	94/98
	IVDE	88	86/88
	IVDP or IVDPs	8	6/8
	HNPE	2	2/2
	Syringomyelia	2	2/2
Anomalous	Atlantoaxial instability (AAI)	1	1/1
Neoplasia		3	2/3
Inflammatory	Immune-mediated	8	8/8
	Myelitis	3	
	SRMA	2	
	Neuritis	2	
	Ganglioneuritis	1	
Vascular	FCEM	1	1/1

^1^ DAMNITV: degenerative, anomalous, metabolic, neoplastic, idiopathic, inflammatory, infectious, toxic, traumatic or vascular. Abbreviation: IVDE, intervertebral disc extrusion; IVDP, intervertebral disc protrusion; FCEM, fibrocartilaginous embolic myelopathy; HNPE, hydrated nucleus pulposus extrusion; SRMA, steroid responsive meningitis-arteritis; Ganglioneuritis, idiopathic bilateral C2 hypertrophic ganglioneuritis.

**Table 5:** Neurological grade vs. resolution of cervical myoclonus.

Neurological Grade	Follow-Up Available	Resolution of CM
Grade 1 (n = 70)	47/70, 67.1%	45/47, 95.7%
Grade 2 (n = 38)	26/38, 68.4%	26/26, 100%
Grade 3 (n = 5)	4/5, 80%	4/5, 80%

## Data Availability

The raw data supporting the conclusions of this article will be made available by the authors on request.

## Abbreviations

The following abbreviations are used in this manuscript:

IVDH	Intervertebral disc herniation
MRI	Magnetic resonance imaging
CM	Cervical myoclonus
IVDE	Intervertebral disc extrusion
EEG	Electroencephalography
EMG	Electromyography
SSM	Spinal segmental myoclonus
SM	Syringomyelia
CT	Computed tomography
IVDD	Intervertebral disc disease
IVDP	Intervertebral disc protrusion
ECVN	European College of Veterinary Neurology
T2W	T2-weighted
T1W	T1-weighted
FLAIR	Fluid-attenuated inversion recovery
SD	Standard deviation
IQR	Interquartile range
CI	Confidence interval
HNPE	Hydrated nucleus pulposus extrusion
MUO	Myelitis/meningomyelitis of unknown origin
SRMA	Steroid responsive meningitis arteritis
AAI	Atlantoaxial instability
FCEM	Fibrocartilaginous embolism
IVD	Intervertebral disc
NSAIDs	Non-steroidal anti-inflammatory
PMMA	Polymethylmethacrylate
